# Association of serum lipid profile and other systemic risk factors with retinal hard exudates in diabetic retinopathy

**DOI:** 10.1007/s10792-024-03263-x

**Published:** 2024-08-02

**Authors:** Harshita Mukesh Hiran, Ajay Kamath, Teena Mariet Mendonca, Gladys R. Rodrigues, Rajesh R. Nayak, Gurudutt Kamath, Sumana J. Kamath

**Affiliations:** 1 Kasturba Medical College Mangalore, Mangalore, India; 2 Manipal Academy of Higher Education, Manipal, India; 3 Department of Ophthalmology, Kasturba Medical College, Mangalore, India

**Keywords:** Diabetes mellitus, Diabetic retinopathy, Dyslipidemia, Diabetic macular edema, Hard exudates

## Abstract

**Purpose:**

Diabetic macular edema is one of the leading causes of vision loss across the world. Hard exudates at the macula can lead to structural abnormalities in the retina leading to irreversible vision loss. Systemic dyslipidemia and other modifiable risk factors when identified and treated early may help prevent substantial vision loss. The purpose of this study was to study the association between serum lipid levels and other systemic risk factors like hemoglobin, HbA1c, and serum creatinine with hard exudates and macular edema in patients with diabetic retinopathy.

**Methods:**

It is a prospective cross-sectional study conducted in a tertiary health care center in South India. 96 patients having diabetic retinopathy with hard exudates were included. Modified Airlie house classification was used to grade the hard exudates. Blood investigations including serum lipid profile, hemoglobin, HbA1c, and serum creatinine were carried out. Central subfield macular thickness was measured using optical coherence tomography.

**Results:**

96 patients of type II DM with diabetic retinopathy were divided into three groups of hard exudates. A statistically significant correlation was observed between the severity of hard exudates and total cholesterol (*p* = 0.00), triglycerides (*p* = 0.00), LDL (*p* = 0.00), and VLDL (*p* = 0.00). HbA1c levels showed a statistically significant correlation with the severity of hard exudates (*p* = 0.09), no significant correlation was noted between hard exudates and hemoglobin levels (*p* = 0.27) and with serum creatinine (*p* = 0.612). A statistically significant association between CSMT and hard exudates (*p* = 0.00) was noted.

**Conclusion:**

In our study, we concluded that the severity of hard exudates is significantly associated with increasing levels of serum total cholesterol, triglycerides, LDL, VLDL, and HbA1c levels in type II DM patients presenting with diabetic retinopathy. The increasing duration of diabetes is significantly associated with increasing severity of hard exudates. Central subfield macular thickness increases with increasing severity of hard exudates in diabetic retinopathy.

**Supplementary Information:**

The online version contains supplementary material available at 10.1007/s10792-024-03263-x.

## Introduction

Diabetic retinopathy, a microangiopathic condition is the most prevalent microvascular consequence of diabetes [1]. One of the hallmark features of non-proliferative diabetic retinopathy is hard exudates which are lipoprotein and macrophage complexes occurring due to leakage from capillaries. Early Treatment Diabetic Retinopathy study shows that dyslipidemia is associated with greater severity of retinal hard exudates [2].

Diabetic macular edema is a leading cause of reduced visual acuity in diabetic retinopathy. Several studies in the literature indicate an association between diabetic macular edema and dyslipidemia [3]. Optical coherence tomography has become the investigation of choice for diabetic macular edema since it is quantifiable and allows for better follow-up [4, 5]. Hard exudates appear as hyperreflective spots in the outer plexiform layer [6].

Structural abnormalities left behind at the exudation site and neuronal degeneration are the reasons for the inability of visual improvement even after the exudates resolve. This risk of irreversible vision loss emphasizes the significance of preventing hard exudates and recognizing the modifiable risk factors [7].

This study aims to study the association between serum lipid levels and other systemic risk factors like hemoglobin, HbA1c, and serum creatinine with hard exudates and macular edema in patients with diabetic retinopathy.

## Methods

A prospective, cross-sectional study was conducted in a tertiary health care center, in South India from October 1st, 2020, to October 31st, 2022.

Before the commencement of the study, the protocol was submitted to the Institutional ethics committee and approval was obtained. Before recruitment, participants were informed about the purpose and anonymity of the study. Prior consent was taken before enrollment in the study.

Patients who presented to the outpatient department with diabetic retinopathy along with hard exudates in the retina were included in the study. Patients with associated vitreoretinal conditions, significant media opacities, and patients already receiving ophthalmic interventions for diabetic retinopathy were excluded from the study. 96 eyes of 96 patients were enrolled in the study. In patients whose eyes fulfilled the inclusion criteria, the eye with the worst vision was considered for the study. Demographic details, Body mass index, type and duration of diabetes mellitus, and history of use of statins were elicited.

**Fig. 1:**
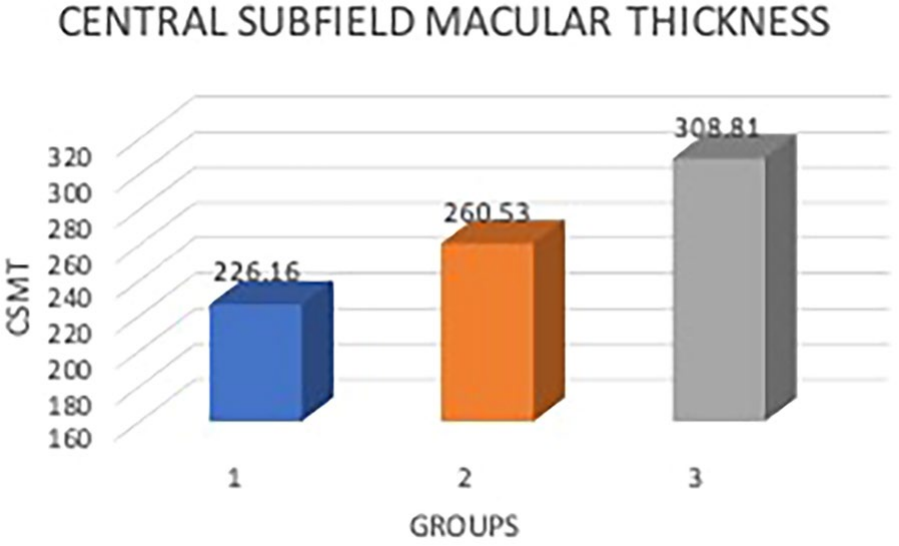
Bar diagram showing mean central subfield macular thickness in each group

All patients underwent comprehensive ocular examination including visual acuity assessment, detailed anterior segment evaluation, intraocular pressure measurement, and fundus examination. Body Mass Index (BMI) was calculated using height and weight measurement. A stereoscopic fundus photograph centered at the macula was obtained using the TRC NW8F fundus camera. The hard exudates on the fundus photo are graded by a single observer using modified Airlie House classification according to ETDRS report 10 [8]. The hard exudates on the fundus photo are graded by a single observer using modified Airlie House classification according to ETDRS report 10 using 30-degree stereoscopic fundus photographs in the standard fields two to seven where grade 0 is absence of hard exudates; grade 1 is a questionable amount of hard exudates; grade 2 is definite hard exudates covering area less than standard image 3; grade 3 is hard exudates more than or equal to standard image 3 but less than image 5; grade 4 is hard exudates more than standard image 5 but less than standard image 4 and grade 5 is hard exudates more than or equal to standard image 4 (Fig. 1). These grades were further divided into three groups where group 1 (absent or minimal hard exudates) included patients with grade 0, 1, or 2 hard exudates; group 2(hard exudates present) included patients with grade 3 or 4 hard exudates and group 3 (prominent hard exudates) included patients with grade 5 hard exudates. Blood investigations were carried out, including serum lipid profile (total cholesterol, triglycerides, LDL, VLDL, and HDL), serum creatinine, hemoglobin, and HbA1c. Optical Coherence Tomography macula scan was done for all the patients to calculate the central subfield macular thickness using Cirrus HD OCT 5000.

The data were analysed using IBM SPSS version 25. The continuous and categorical data within each group was analysed using descriptive statistics. The continuous variables were compared among the three groups using ANOVA, and post-hoc analysis was used to check for individual inter-group differences. Correlation between central subfield macular thickness and other continuous variables was done using Pearson’s correlation for continuous variables. A *p* value of < 0.05 was considered significant for all analyses.

## Results

A total of 96 eyes of 96 type 2 DM patients were included in the study, with 32 participants in each group of hard exudates. The mean age among the 96 participants was 58.61 ± 8.11 years. Group-wise distribution showed the mean age in group one was 60.69 ± 9.865 years, the mean age in group two was 57.91 ± 7.63 years, and the mean age in group three was 57.25 ± 6.242 years. Out of the 96 participants, 64 (66.67%) were male, and 32 (33.33%) were females. The mean BMI of 96 participants was 25.89 ± 2.69 kg/m^2^. The mean BMI in group one was 25.71 ± 2.34 kg/m^2^, 25.83 ± 3.19 kg/m^2^ in group two, and 26.12 ± 2.53 kg/m^2^ in group three. The mean duration of diabetes among 96 participants was 10.47 ± 6.96 years. The mean duration of diabetes in group one was 8.39 ± 5.98 years; in group two mean duration of diabetes was 11.17 ± 7.68 years, and 11.87 ± 6.82 years in group three.

The correlation between the duration of diabetes and the grouping of hard exudates was studied using the Spearman correlation as the duration of diabetes is a continuous variable and the grouping of hard exudates an ordinal variables [9]. A statistically significant positive correlation between the duration of diabetes and the grouping of hard exudates was seen with greater severity of exudates with a longer duration of diabetes (*p* = 0.045).

Grade one (n = 4, 12.5%) and grade two (n = 28, 87.5%) were included in group one; grade three (n = 19, 59.4%) and grade four (n = 13, 40.6%) were included in group two, and grade five (n = 32,100%) were included in group three according to the ETDRS classification.

Comparison of the continuous variables was made between the three groups categorized based on the severity of hard exudates using ANOVA IBM SPSS VERSION 25 to study the relationship between the three groups of hard exudates with central macular thickness, hemoglobin, HbA1c, Lipid profile (Total cholesterol [TC], triglycerides [TG], Low-density lipoprotein [LDL], Very low-density lipoprotein [VLDL], high-density lipoprotein [HDL]) and serum creatinine (Table 1). Tukey’s post hoc comparison of significant variables between each of the three groups revealed that CSMT and Total cholesterol values were greater for group three followed by group two and group one; HbA1c values were lesser for group one than group two and three; Triglycerides, LDL, and VLDL values were significantly greater in group three compared to the other two groups (Table 2).

**Table 1:** Table showing the correlation between central subfield macular thickness and continuous variables using Pearson’s coefficient

	Variable	Group	Mean ± SD	*p* value
A	CSMT (µm)	1 (n = 32)	226.16 ± 33.98	0.000
		2 (n = 32)	260.53 ± 58.26	
		3 (n = 32)	308.81 ± 71.51	
B	Hemoglobin (gm/dl)	1 (n = 32)	13.05 ± 1.623	0.274
		2 (n = 32)	12.49 ± 1.34	
		3 (n = 32)	12.64 ± 1.275	
C	HbA1c (%)	1 (n = 32)	7.65 ± 2.669	0.009
		2 (n = 32)	9.14 ± 2.35	
		3 (n = 32)	9.31 ± 1.81	
D	Total cholesterol(mg/dl)	1 (n = 32)	163.03 ± 28.06	0.000
		2 (n = 32)	190.06 ± 33.78	
		3 (n = 32)	244.25 ± 62.49	
E	Triglycerides (mg/dl)	1 (n = 32)	130.65 ± 35.51	0.000
		2 (n = 32)	161.46 ± 59.84	
		3 (n = 32)	239.85 ± 125.98	
F	LDL (mg/dl)	1 (n = 32)	86.19 ± 33.97	0.000
		2 (n = 32)	110.06 ± 37.70	
		3 (n = 32)	139.01 ± 56.35	
G	VLDL (mg/dl)	1 (n = 32)	28.62 ± 11.38	0.000
		2 (n = 32)	34.36 ± 16.81	
		3 (n = 32)	56.37 ± 32.03	
H	HDL (mg/dl)	1 (n = 32)	45.39 ± 8.61	0.684
		2 (n = 32)	44.17 ± 10.21	
		3 (n = 32)	43.51 ± 7.137	
I	Serum Creatinine(mg/dl)	1 (n = 32)	0.89 ± 0.38	0.612
		2 (n = 32)	0.96 ± 0.33	
		3 (n = 32)	0.97 ± 0.32

**Table 2:** Table showing Tukey’s post hoc comparison of significant variables between each of the three groups

	Variable	Group 1	Group 2	Mean difference	*p* value
A	CSMT(µm)	1	2	− 34.375	0.045
		1	3	− 82.65	0.000
		2	3	− 48.28	0.003
B	Hemoglobin (gm/dl)	1	2	0.55	0.266
		1	3	0.40	0.490
		2	3	− 0.15	0.907
C	HbA1c (%)	1	2	− 1.48	0.031
		1	3	− 1.65	0.014
		2	3	− 0.16	0.954
D	Total cholesterol(mg/dl)	1	2	− 27.03	0.042
		1	3	− 81.21	0.000
		2	3	− 54.18	0.000
E	Triglycerides (mg/dl)	1	2	− 30.81	0.304
		1	3	− 109.20	0.000
		2	3	− 78.38	0.001
F	LDL (mg/dl)	1	2	− 23.87	0.080
		1	3	− 52.82	0.000
		2	3	− 28.95	0.026
G	VLDL (mg/dl)	1	2	− 5.73	0.549
		1	3	− 27.75	0.000
		2	3	− 22.01	0.000
H	HDL (mg/dl)	1	2	1.22	0.841
		1	3	1.88	0.666
		2	3	0.65	0.952
I	Serum Creatinine(mg/dl)	1	2	− 0.07	0.704
		1	3	− 0.079	0.635
		2	3	− 0.009	0.993

In group one, the mean CSMT obtained on OCT was 226.16 ± 33.98 µm as compared to 260.53 ± 58.26 µm in group two and 308.81 ± 71.51 µm in group three with a *p* value of 0.00 showing that the difference in the CSMT values among the three groups was statistically significant (Fig. 1).

The mean hemoglobin in group one was 13.05 ± 1.623 gm/dl, 12.49 ± 1.34 gm/dl in group two, and 12.64 ± 1.275 gm/dl in group three, with a *p* value of 0.274. Thus, the difference in the hemoglobin levels of the three groups was not statistically significant.

**Fig. 2:**
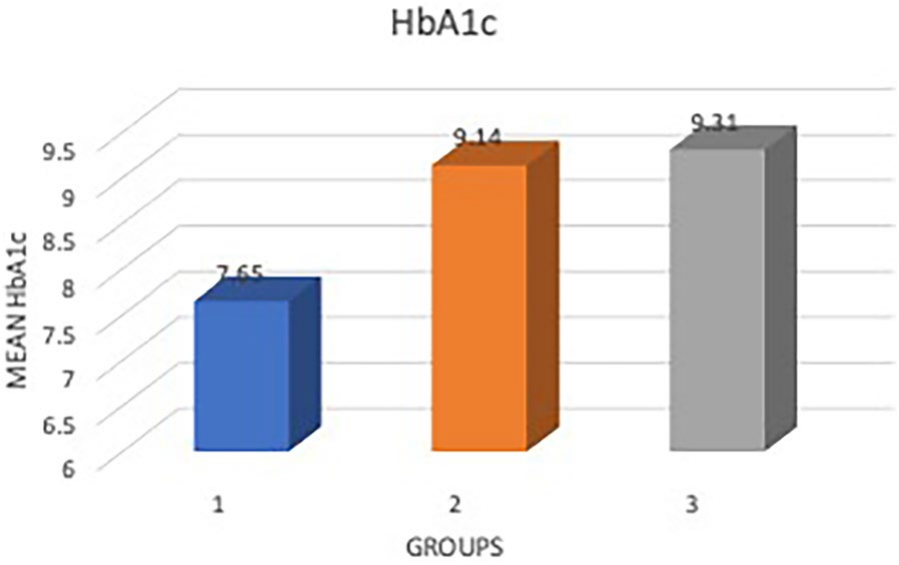
Bar diagram showing the mean HbA1c among the three groups

The mean HbA1c in group one was 7.65 ± 2.669% compared to 9.14 ± 2.35% in group two and 9.31 ± 1.81% in group three, with a *p* value of 0.009 showing a statistically significant difference among the three groups. The post hoc comparison among the three groups for HbA1c showed lesser values for group one than for groups two and three (Fig. 2).

The mean total cholesterol in group one was 163.03 ± 28.06 mg/dl as compared to 190.06 ± 33.78 mg/dl in group two and 244.25 ± 62.49 mg/dl in group three with a *p* value of 0.00 showing a statistical significant difference in the total cholesterol values among the three groups. Post hoc analysis of total cholesterol levels among the three groups showed values greater for group three, followed by groups two and one.

The mean triglyceride levels in group one was 130.65 ± 35.51 mg/dl compared to 161.46 ± 59.84 mg/dl in group two and 239.85 ± 125.98 mg/dl in group three with a *p* value of 0.00 showing a statistically significant difference in the triglyceride levels among the three groups. Post hoc analysis of the triglyceride levels among the three groups showed significantly greater values in group three compared to the other two groups.

The mean LDL levels in group one was 86.19 ± 33.97 mg/dl compared to 110.06 ± 37.70 mg/dl in group two and 139.01 ± 56.35 mg/dl in group three, with a *p* value of 0.00 showing a statistically significant difference in the LDL levels among the three groups. Post hoc analysis of the LDL levels among the three groups showed values were significantly greater in group three compared to the other two groups.

The mean VLDL levels in group one was 28.62 ± 11.38 mg/dl compared to 34.36 ± 16.81 mg/dl in group two and 56.37 ± 32.03 mg/dl in group three with a *p* value of 0.00 showing a statistically significant difference in the VLDL levels among the three groups. Post hoc analysis of the VLDL levels among the three groups showed values were significantly greater in group three compared to the other two groups.

**Fig. 3:**
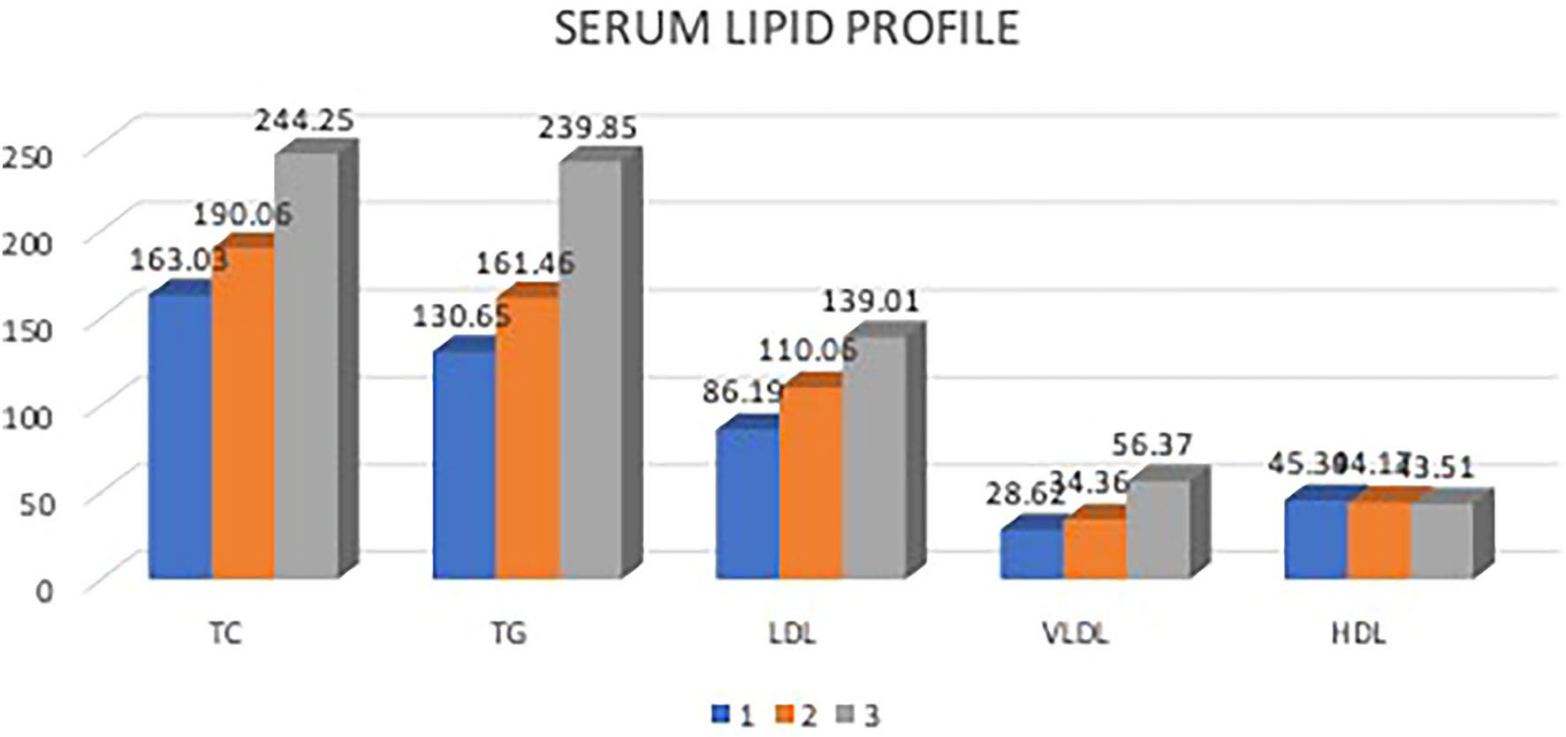
Bar graph showing the mean serum lipid profile values among the three groups

The mean HDL levels in group one were 45.39 ± 8.61 mg/dl compared to 44.17 ± 10.21 mg/dl in group two and 43.51 ± 7.137 mg/dl in group three, with a *p* value of 0.684 showing no statistically significant difference in the HDL levels among the three groups. Multiple bar diagram showing the mean levels of total cholesterol, triglycerides, LDL, VLDL and HDL among the three groups seen in Fig. 3.

The mean creatinine levels in group one was 0.89 ± 0.38 mg/dl compared to 0.96 ± 0.33 mg/dl in group two and 0.97 ± 0.32 mg/dl in group three with a *p* value of 0.612 showing no statistically significant difference in creatinine levels among the three groups.

**Fig. 4:**
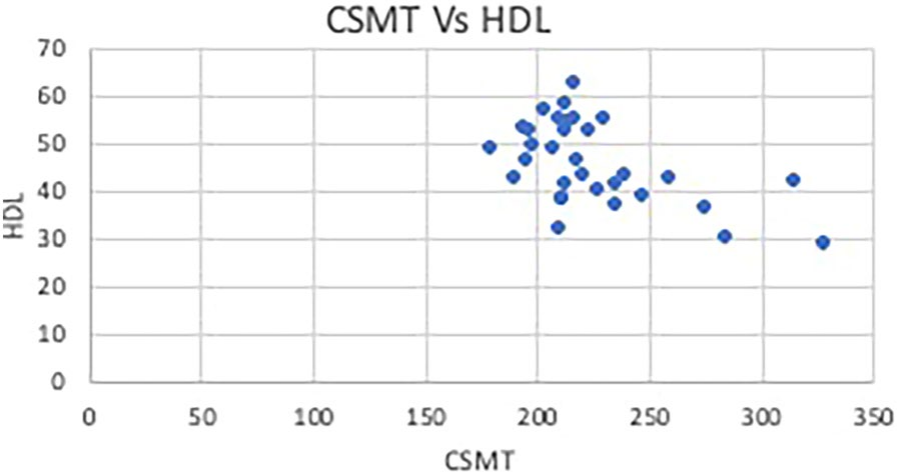
Scatter diagram showing Pearson’s correlation between CSMT and HDL in group 1 showing a significant negative correlation

In group one, two participants were on statins (6.3%) as compared to eight participants in group two (25%) and two participants in group three (6.3%). The correlation between central subfield macular thickness and hemoglobin, HbA1c, lipid profile, and serum creatinine was analyzed using Pearson’s correlation among the three groups. (Pearson’s correlation coefficient = r) (Table 3). The correlation showed that within group one, there was a statistically significant negative correlation between CSMT and HDL (*p* = 0.001) (Fig. 4).

**Table 3:** Table showing the correlation between central subfield macular thickness and continuous variables using Pearson’s coefficient

Group	r/p	Variable 1	Variable 2							
			Hemoglobin	HbA1c	TC	TG	LDL	VLDL	HDL	S.Creatinine
1	r	CSMT(µm)	0.217	0.328	− 0.199	− 0.097	0.293	0.164	− 0.545	0.261
	P		0.233	0.067	0.276	0.598	0.103	0.370	0.001	0.149
2	r	CSMT(µm)	− 0.091	0.093	0.185	0.212	0.119	0.053	− 0.149	0.152
	P		0.619	0.614	0.310	0.244	0.517	0.775	0.417	0.407
3	r	CSMT(µm)	− 0.309	0.153	0.160	− 0.138	0.104	− 0.237	0.035	− 0.101
	P		0.086	0.404	0.382	0.451	0.571	0.191	0.849	0.582

## Discussion

The demographic characteristics of our study showed a mean age of 58.61 ± 8.11 years of which 64 (66.67%) were males, and 32 (33.33%) were females; mean BMI was 25.87 ± 2.69 kg/m^2^(overweight class) and the mean duration of diabetes was 10.47 ± 6.96 years. A similar study by Sasaki et al. measuring the association between hard exudates and serum lipid profile found a mean age of 60.5 years, with 70.9% of patients being male, and the mean BMI was 31.1 kg/m^2^, the mean duration of diabetes was 16.7 years [10].

In the present study, we found no statistically significant correlation between the grade of hard exudates and hemoglobin levels (*p* = 0.27). A similar prospective study done by Mohan VK et al. showed a statistically significant correlation between low hemoglobin levels and hard exudate levels in diabetic retinopathy [11]. However, in their study, the mean Hemoglobin levels(+ SD) in patients with and without diabetic retinopathy were 11.24 (+ 2.1) g/dl and 12.71 (+ 2.1) g/dl, respectively. The authors have not defined anemia in their study. The mean levels of hemoglobin in both groups were comparable with those of our study and were not clinically significant.

In this study, we found that mean HbA1c levels show a statistically significant correlation with the grades of hard exudates (*p* = 0.09), suggesting that higher HbA1c levels contribute to increasing retinal hard exudates in diabetic retinopathy. These findings were consistent with a study carried out by Silpa et al. on 242 patients in Kerala, India [12].

In the present study, the correlation between the grading of hard exudates and serum lipid profile was studied, and a statistically significant correlation was observed with total cholesterol (*p* = 0.00), triglycerides (*p* = 0.00), LDL (*p* = 0.00) and VLDL (*p* = 0.00) with post hoc analysis showing greater values in group three followed by group two and one. There was no correlation seen with HDL levels (*p* = 0.684). This suggests that the increased severity of hard exudates is associated with dyslipidemia.

A similar study by Idiculla J et al. on type II diabetics who presented with hard exudates in the retina showed a significant correlation between dyslipidemia, total cholesterol levels, LDL levels, and hard exudate formation. No significant association was noted with HDL, and triglyceride levels showed a trend toward significance [13]. These findings were consistent with our study.

A study done under ETDRS suggested that raised total serum cholesterol or LDL increases the likelihood of developing hard exudates by two times, and the level of hard exudates is also associated with the risk of vision loss [2].

CURES eye study in Chennai showed a significant association of diabetic retinopathy with triglyceride levels and of LDL cholesterol with diabetic macular edema [14].

Another variable evaluated in this study was the association between serum creatinine and the severity of hard exudates, and no significant association was seen in our research (*p* = 0.612). This was discordant with a similar study by Sachdev et al., which showed a statistically significant association between hard exudates and serum creatinine [15]. This discrepancy could be due to variability in sample size as the sample size in their study was 180; nearly double that of our study. The mean duration of DM can play a significant role in the occurrence of higher serum creatinine levels. Since the authors have not specified the mean duration of DM in their study it is hard to conclude.

Another objective of this study was to measure the CSMT on SD-OCT and evaluate its association with hard exudates severity and various risk factors like serum lipid profile, hemoglobin, HbA1c, and serum creatinine. A study by Browning et al. showed that CSMT is the advised measurement on OCT for DME as it measures the central macula as compared to Total Macular Volume [16]^.^ We found a statistically significant association between CSMT and hard exudates (*p* = 0.00), showing increasing CSMT with increasing levels of hard exudates. A negative correlation between CSMT and HDL in group one of hard exudates (*p* = 0.001). No significant correlation was noted between CSMT and hemoglobin, HbA1c, serum creatinine, total cholesterol, triglyceride, LDL, and VLDL levels.

A similar study by Sasaki et al. found that raised LDL levels were associated with higher CSMT and CSMV in diabetics without diabetic macular edema, which is discordant with our study [10].

Davoudi et al. carried out a similar study in type II diabetic patients measuring the CSMT, CSMV, and TMV and found a significant correlation between total macular volume and raised TC and triglycerides and no association between HDL cholesterol and total macular volume [17].

Increased deposition of hard exudates leads to an increasing risk of subretinal fibrosis which can lead to irreversible visual loss. Prior studies have suggested that hard exudate deposition can lead to photoreceptor and neuron layer degeneration in the OPL thus leading to disturbed central visual acuity, reduced fixation, and thickening in the fovea [18].

This study demonstrates an association between hard exudates in DR and dyslipidemia. Studies have also shown that using statins reduces the severity of hard exudates in DR [19]. Thus, our study highlights the importance of including serum lipid profiles during routine evaluation in diabetic retinopathy patients presenting with hard exudates to prevent irreversible visual loss.

There are a few limitations of this study. The sample size was small and follow-up studies are required. In this study, the eye with the worst visual acuity was included hence, the presence of diffuse DME may be a confounding factor. The correlation between clinical grading of CSME and lipid profile was not studied. The association between the use of statins with the severity of retinal hard exudates was not studied. 

## Conclusion

In our study, we concluded that the severity of hard exudates is significantly associated with increasing levels of serum total cholesterol, triglycerides, LDL, VLDL, and HbA1c levels in type II DM patients presenting with diabetic retinopathy. The increasing duration of diabetes is significantly associated with increasing severity of hard exudates. Central subfield macular thickness on SD-OCT increases with increasing severity of hard exudates in DR. Central subfield macular thickness shows a negative correlation with HDL levels in the mild grade of hard exudates in diabetic retinopathy. Hence this study highlights the importance of strict control of serum lipid levels along with glycaemic control in diabetic retinopathy.

## Supplementary Information

Below is the link to the electronic supplementary material.

Supplementary file1 (XLSX 20 kb)
